# Bayat-driven FOPID controller design for biogas-based microgrid with real-time validation

**DOI:** 10.1038/s41598-025-20883-0

**Published:** 2025-10-22

**Authors:** T. K. Bashishtha, V. P. Singh, Tarun Varshney, Sanjeevikumar Padmanaban

**Affiliations:** 1 Dept. of Electrical Engineering, Malaviya National Institute of Technology, Jaipur, 302017 Rajasthan India; 2 Dept. of EECE, SSES, Sharda University, Greater Noida, UP India; 3 Department of Electrical Engineering, IT and Cybernetics, University of South-Eastern Norway, Porsgrunn, Norway

**Keywords:** Fractional Order PID, Bayat method, Decentralized Microgrid, Biogas Generator, Real-Time Simulation, Frequency Control, Energy science and technology, Engineering

## Abstract

The energy policies of the $21^{st}$ century are increasingly focused on promoting generation solutions with minimal environmental impact. In response to strategic initiatives, the accelerating depletion of fossil fuel reserves has led to integrating renewable sources for power generation. The uncertain nature of solar and wind energy sources, along with fluctuating load demands, leads to frequency instability. This study addresses the challenge of frequency instability by designing a Bayat-tuned fractional-order proportional-integral-derivative (FOPID) controller for a decentralized microgrid $(Dz \mu G)$. The proposed $Dz \mu G$ model consists of environmentally friendly energy sources such as a biogas turbine generator (BTG), a biodiesel engine generator (BEG), other distributed generation units (DGUs), and energy storage devices (ESDs). The mathematical modeling of $Dz \mu G$ components is carried out using first-order transfer functions, which are combined to derive the overall transfer function of $Dz \mu G$ model. This composite model is then approximated as a first-order plus time delay (FOPTD) system to simplify FOPID controller design. The parameters of the FOPID controller are optimized using the Bayat method to achieve robust performance under set-point tracking (SPT) and load disturbance rejection (LDR) scenarios. Based on this approach, three controller variants i.e., FOPID-$Bayat_{SP1.4}$, FOPID-$Bayat_{SP2.0}$, and FOPID-$Bayat_{LD1.4}$, are developed. To validate the effectiveness of the proposed control strategy, various simulation scenarios are considered, including load disturbances and varying levels of solar and wind power penetration. The performance of the controllers is evaluated in terms of frequency deviation, error mitigation, and transient behavior under SPT and LDR conditions. A comparative analysis using error indices, time-domain metrics, control effort, and frequency plots confirms the effectiveness of the Bayat-tuned FOPID designs. Furthermore, real-time validation using the OPAL-RT simulator underscores their practical potential in maintaining frequency stability within $Dz \mu G$ systems. Owing to the performance analysis, it is justified that discussed *FOPID–Bayat* controllers consistently ensured controllability with a minimum rise time of $4.02 \times 10^{-5}\,\text {s}$, a nearly constant settling time of $\sim 49.8\,\text {s}$, and reduced control effort down to 0.12. Furthermore, error index evaluation confirmed that *FOPID–Bayat*$_{SP2.0}$ outperformed other configurations by achieving the lowest IAE (8.737), ITAE (223.0), ITSE (40.39), and ISE (1.706), thereby demonstrating superior efficiency and robustness.

## Introduction

### Motivation and background

In the $21^{st}$ century, energy policies increasingly prioritize sustainable generation methods that minimize environmental impact^1^. This shift is driven by the dual pressures of strategic energy planning and the accelerating depletion of fossil fuel resources, both of which have significantly increased global interest in renewable energy sources (RESs) as alternatives to conventional power generation^2^. Traditional energy systems face several inherent challenges, including high levels of pollution, complex and rigid network infrastructures, and economic inefficiencies due to considerable transmission and distribution losses. These drawbacks have catalyzed the development of microgrids as a more adaptable and sustainable solution.

Microgrids, particularly in remote or off-grid regions, offer a practical and cost-effective alternative where extending the main grid is neither feasible nor economical. By integrating RESs, microgrids address the limitations of conventional systems while offering several operational advantages such as reduced transmission losses, improved system reliability and security, enhanced scalability, and more flexible control architectures. Furthermore, the use of RESs significantly reduces the environmental footprint of power generation, helping to mitigate the negative effects associated with fossil fuel consumption^3^. Beyond environmental benefits, RES integration also presents opportunities for significant cost savings and improved energy resilience, making microgrids a promising component of modern energy systems.

### Literature on microgrid models

A decentralized microgrid ($Dz\mu G$) typically comprises both controlled and uncontrolled sources, spanning conventional and renewable technologies, along with energy storage systems and dynamic loads^4^. The primary objective of a $Dz\mu G$ is to coordinate and optimize the operation of its diverse generation units and storage systems to reliably meet continuously fluctuating load demands^5^. In response to rising energy demands and growing environmental and economic concerns, there is a notable trend toward integrating more renewable (non-conventional) sources in place of conventional ones.

**Table 1:** Literature available on $Dz\mu G$ architecture and controller.

Ref.	Renewable sources	Controllble sources	Controller	BESS	FESS	Other storage
^6^	SPV, WTG	MT, FC, DEG	IPD-(1+I)	✓	✓	✗
^7^	SPV, WTG	Thermal	PI	✗	✗	✗
^8^	SPV, WTG	Thermal unit	Fuzzy-PID	✓	✓	✗
^9^	WTG, SPV	DEG, MTG, FC	PID	✓	✓	✗
^10^	SPV, WTG	FC, DEG, MT	Multi-stage PID	✓	✓	✗
^11^	SPV, WTG	DEG, FC	PD-PI	✓	✗	PEV
^12^	SPV, WTG	AE-FC, DEG	PD-PI	✓	✓	✗
^13^	SPV, WTG	BEG, BTG	Fuzzy-PID	✓	✓	✗
^14^	WTG, SPV	FC, DEG	PD-(1+PI)	✓	✗	✗
^15^	SPV, WTG	MT, FC, DEG	PID	✓	✓	✗
^16^	SPV	BEG, BTG	I, PI, PD, PID, PIDF	✓	✗	✗
^17^	SPV, SWE	BGT, PEMFC	PI(1+PD)	✓	✓	SMES
^18^	SPV, WTG	AE-FC, DEG, MT	PID	✓	✓	✗
^19^	SPV, WTG	AE-FC, DEG, MT	PI	✓	✓	UC
^20^	SPV, WTG	AE-FC	fuzzy PID	✗	✗	UC, EV
^21^	SPV, WTG	DEG	PD-PI	✗	✗	PHEV
^22^	SPV, WTG	-	PID	✓	✗	✗
^23^	SPV, WTG	BGT, BEG, Hydro	PI, PID, PIDF	✓	✗	✗
^24^	SPV, WTG	DEG	2-DOF-PID	✓	✓	EV
^25^	SPV, WTG	DEG	Fuzzy-PI	✓	✗	✗
Proposed	SPV, WTG	DEG, MTG, BEG, BTG	FOPID	✓	✓	UC, EV

As per literature^8^, solar photovoltaic (SPV) systems and wind turbine generator (WTG) serve as typical uncontrolled renewable sources, while battery energy storage systems (BESS) and flywheel energy storage systems (FESS) are widely used to store surplus power. Diesel engine generator (DEG), on the other hand, are commonly employed as controlled sources to compensate for power deficits^26^. Some models also incorporate thermal power plants alongside SPV, WTG, BESS, and FESS to diversify generation options^7,8^.

Several microgrid configurations have been proposed in the literature^27–29^, showcasing different combinations of controlled sources and storage devices. In more advanced architectures, fuel cells (FCs) are integrated with SPV, WTG, DEG, BESS, and FESS, serving as controllable units capable of utilizing surplus renewable energy via aqua-electrolyzers to produce hydrogen for later use^10,30,31^. Other models explore the use of aqua-based fuels for power generation, supplementing traditional RESs and storage devices with components such as microturbine generators (MTGs) and DEGs to enhance system robustness^9,10,12,32,33^. MTGs are particularly valuable for frequency regulation and improving power supply stability^28,34^. Furthermore, electric vehicles (EVs) are gaining attention as mobile energy storage units that can dynamically adjust their charging and discharging patterns in response to real-time load fluctuations^34^. 

The literature suggested that most of the researchers considered SPV, WTG, as uncontrollable sources, DEG, and MTG as controllable sources, and BESS, FESS for storage purposes. As per the author’s knowledge, biogas turbine generator (BTG) and biodiesel engine generator (BEG) are not utilized as controllable units with SPV, WTG, DEG, MTG, BESS, FESS, EV for frequency stability of $Dz \mu G$. Integrating BTG and DEG in $Dz \mu G$ enhanced its reliability and made the environment eco-friendly, as these power generation units uses waste products for power generation. This is one of the main contributions of this article.

Despite the growing interest in decentralized microgrid systems, ensuring frequency stability under dynamic conditions remains a critical challenge, particularly in the presence of load disturbances and renewable energy intermittencies. The frequency stability of $Dz \mu G$ are maintained by keeping a proper balance between generation and load. As load and power generation through renewable sources are variable, it leads to the issue of load frequency control (LFC). LFC is managed with the help of storage units and additional controlled sources of conventional and non-conventional nature^35^. To regulate power of controlled sources, various control schemes such as PD-PI^11^ IPD-(1+I)^6^, PD-(1+PI)^14^, fuzzy PIDF^36^, fuzzy PI-PD^37^, type II fuzzy^38^ and PID^39,40^, FOPI^41^ are incorporated into $Dz \mu G$ as secondary control schemes to manage LFC. The detailed summary of microgrid schema with their control design is provided in Table 1. Conventional PID controllers often fall short in handling the complex dynamics and uncertainties inherent in such systems. Modified controllers are discussed in the literature^34,42,43^. Sahu et. al in^43^ presented an efficient control technique for an islanded microgrid of two area. Authors have designed type-II fuzzy based PID controller to maintain the tie-line power and frequency within the limits by incorporating various uncertainties. For optimal tuning of gain of PID controller, improved salp swarm optimization as an powerful algorithm has been implemented. Authors have also performed the comparative analysis for the designed controller with type-I fuzzy controller while outcomes of optimization algorithm have been compared with other popular algorithms. Furthermore, a fractional order PI controller is implemented by Sahu et al. in^34^, by utilizing type2 fuzzy approach for maintaining the power flow as well as frequency of a complex multi-area microgrid considering various disturbances. Authors have used cow search algorithm for the optimum tuning of the controller parameters. In addition to that authors have performed comparative study validate the superiority of designed controller over generalised type-2, PID based on fuzzy type-1 and PID controllers.

Moreover, the issue related with the frequency fluctuation in a renewable and EV integrated microgrid architecture, is taken into account^44^. Author has developed a fractional order control scheme as power system stabilizer based on fuzzy technique tuned with advanced sine cosine algorithm for reducing the duration of settling time of frequency deviation. In addition to the approaches in the segment of regulating the frequency, Bhatta et. al in^45^ have proposed a fractional order based controller utilizing type 2 fuzzy technique to frequency and voltage profile of two area microgrid facilitated with various renewable integration having different uncertainties. In the aforementioned approach, author has exploited an algorithm based on Quassi- oppositional path finderfor the optimum selection of controller parameters.

Fractional-order PID (FOPID) controllers effectively deal with complex dynamics and system uncertainty, and offer enhanced flexibility with improved performance. In literature, there is a lack of comprehensive studies applying systematic tuning methods such as the Bayat method to FOPID controllers for decentralized microgrids. Moreover, the suitability of different Bayat tuning configurations for specific control objectives like set-point tracking and load disturbance rejection remains underexplored.

This research is motivated by the need to fill this gap by investigating the application of the Bayat tuning method for designing FOPID controllers tailored to a $Dz \mu G$ model. This study aims to develop an effective control strategy that not only stabilizes frequency but also optimizes transient performance and control effort for a $Dz \mu G$ model.

A key contribution of this work lies in the novel integration of Bayat methodology-based tuning, First Order Plus Time Delay (FOPTD) system modeling, and real-time validation, which has not been explored previously in the context of microgrid frequency regulation. While each of these elements has been studied independently in prior research, their combined application offers a comprehensive framework that bridges theoretical controller design and practical implementation. This synergistic approach enhances the robustness and reliability of fractional-order PID (FOPID) controllers under dynamic operating conditions, demonstrating a clear advancement in the field of intelligent control for renewable-integrated microgrids.

### Contribution and highlights of study

The major contribution of this article is the investigation of Bayat assisted FOPID controller for enhancing frequency stability of $Dz\mu G$ model. However, our contribution lies in applying Bayat method under set-point and load disturbance scenarios to design an FOPID controller that can maintain the frequency stability of a biogas-based Dz$\upmu$G. The FOPID controller efficacy in managing the frequency stability is validated through OPAL-RT. To implement FOPID controller, the linear transfer function of the $Dz\mu G$ model is derived by linearizing and approximating the system as a first-order plus time delay (FOPTD) model. The FOPID controller is then designed for both set-point tracking and load disturbance rejection modes. A comparative analysis of various FOPID controller variants including FOPID-$Bayat_{SP1.4}$, FOPID-$Bayat_{SP2.0}$, and FOPID-$Bayat_{LD1.4}$ is conducted to determine the most effective control design for maintaining frequency stability of $Dz\mu G$. The effectiveness of this tuning method is validated through detailed evaluations of transient responses, control signal magnitudes, and frequency deviation profiles under SPT, LDR and variations in load, solar and wind power. Furthermore, the presented tabulated data of parametric sensitivity and real time validation support the robustness and suitability of the FOPID control designs for achieving frequency stability of $Dz\mu G$. The significant contributions of the presented article are summarized as follows:

- Developed a novel architecture for decentralized Microgrid ($Dz \mu G$) incorporating eco-friendly generation sources, including a biogas turbine generator and a biodiesel engine generator, along with other Distributed Generation Units (DGUs) and Energy Storage Devices (ESDs).
- Designed a fractional order proportional-integral-derivative (FOPID) controller to manage frequency deviations and frequency stability of $Dz\mu G$, utilizing a first-order model-based mathematical representation of the $Dz \mu G$.
- Implemented the Bayat method for both set point tracking and load disturbance rejection scenarios to determine FOPID controller parameters, aiming to maintain frequency stability of $Dz \mu G$ model.
- Ensured robustness of the FOPID controller for frequency regulation by performing parametric sensitivity analysis.
- Evaluated the performance of the various variations of the FOPID controller to maintain frequency stability during multiple load variations, and variations in solar and wind power, focusing on frequency error mitigation, with minimal controller effort, and attaining a feasible transient response.
- Validated the effectiveness of FOPID controllers i.e., FOPID-$Bayat_{SP1.4}$, FOPID-$Bayat_{SP2.0}$, and FOPID-$Bayat_{LD1.4}$, through real-time simulation using the OPAL-RT platform, demonstrating successful frequency error mitigation for the $Dz \mu G$ model.

### Organization of the study

The outline of this article is structured as follows. Section II deals with architecture and equivalent modeling of decentralized Microgrid ($Dz \mu G$). Further, approximation based modelling of $Dz \mu G$ is discussed in section III. Section IV contains FOPID controller design approach and Bayat tuning method. Section V presents results and discussion utilizing Bayat tuning method under SPT and LDR scenarios. Various analyses and main findings are also provided in this section. Further, the concluding remarks with future directions are presented in section VI.

## System under study: decentralized microgrid

**Fig. 1:**
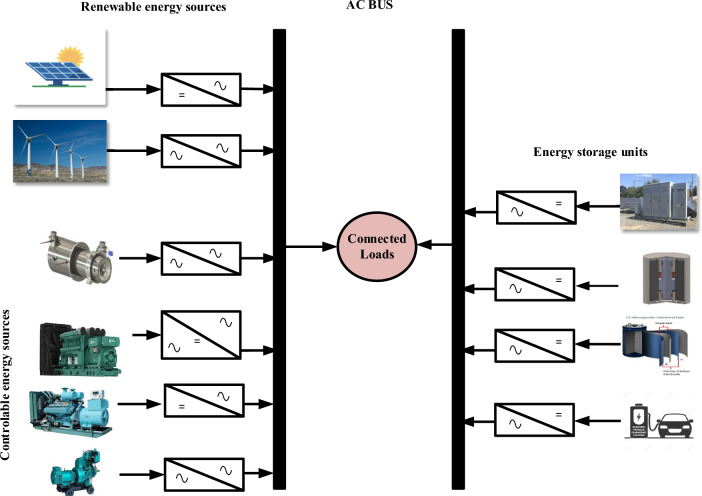
Schema of decentralized microgrid.

The generalized schema of a decentralized microgrid $(Dz \mu G)$ is shown in Fig. 1. The presented model is categorized into three categories, i.e., renewable energy sources, controllable sources, and energy storage devices. In Fig. 1, PV and WTG are renewable energy sources, and DEG, BEG, BTG, and MTG are controllable sources. Moreover, BESS, FESS, UC, and EV are considered as storage units. The main aim of these components is to meet the load demand of the connected load. In Fig. 1 all components are equipped with various power electronic based converters to convert power from AC to DC and vice -versa.

**Fig. 2:**
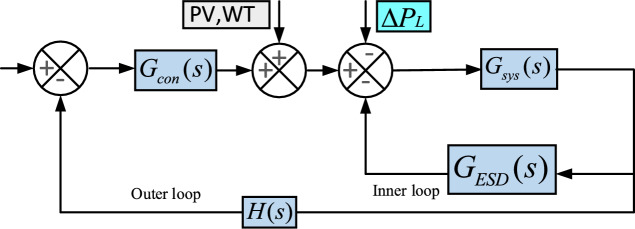
Closed loop based modelling of $Dz\mu G$ components.

The block diagram illustrating microgrid model in in Fig. 1 is further elaborated into closed loop based modelling of microgrid components, which are deicted in Fig. 2. It consists system dynamics ($G_{sys}$), controllable sources ($G_{con}$), energy storage devices ($G_{ESD}$), and a unity feedback. The mathematical expression for $G_{sys}$, $G_{ESD}$ and $G_{con}$ can be expressed in (1)-(3). These models are further classified into two control loops, i.e, primary and secondary loops. $G_{sys}$ and $G_{ESD}$ are main components of primary loop and form a inner closed loop, which is expressed as $G_{inner}$, where as $G_{con}$ is the forward path gain, which is the integral part of secondary loop and connected in series with $G_{inner}$.

1 $$\begin{aligned} G_{con}= & \frac{K_{DEG}}{1+sT_{DEG}}+ \frac{K_{BEG}}{1+sT_{BEG}}+\frac{K_{BTG}}{1+sT_{BTG}}+\frac{K_{MTG}}{1+sT_{MTG}} \end{aligned}$$

2 $$\begin{aligned} G_{ESD}= & \frac{K_{BESS}}{1+sT_{BESS}}+ \frac{K_{FESS}}{1+sT_{FESS}}+\frac{K_{UC}}{1+sT_{UC}}+\frac{K_{EV}}{1+sT_{EV}} \end{aligned}$$

3 $$\begin{aligned} G_{sys}= & \frac{1}{D+Ms} \end{aligned}$$

A detailed analysis to obtain an equivalent transfer function is as follows.

4 $$\begin{aligned} G_{inner}(s)=\frac{G_{sys}(s)}{1+G_{sys}(s)G_{ESD}(s)} \end{aligned}$$

Considering the feedback path as unity, an equivalent model of $Dz \mu G$ i.e. $\hat{G_{Dz \mu G}(s)}$ is formed.

5 $$\begin{aligned} \hat{G_{Dz \mu G}(s)} =\frac{G_{inner}(s)G_{con}(s)}{1+G_{inner}(s)G_{con}(s)H(s)} \end{aligned}$$

Utilizing the (4) and (5), the obtained equivalent transfer function of $Dz \mu G$ model is expressed in (6).

6 $$\begin{aligned} \begin{aligned} \hat{G_{Dz\mu G}(s)}=\dfrac{\begin{array}{r} {0.06949 s^7+1.699s^6 + 13.66s^5+42.05s^4+}\\ { 62.96s^3 + 49.31s^2 +19.38s +3.003}\end{array}}{\begin{array}{r} {0.008791s^9 +0.2272s^8 +2.053s^7 + 8.348s^6 + 20.52s^5}\\ { +33.9s^4 +36.97 s^3 + 24.66 s^2 + 8.909 s +1.318}\end{array}} \end{aligned} \end{aligned}$$

**Fig. 3:**
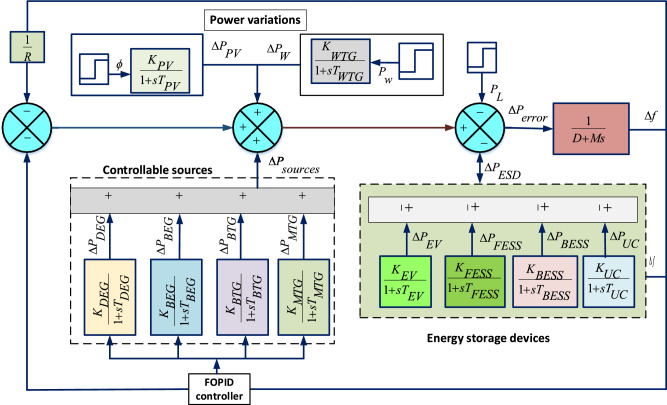
Transfer function model.

The closed loop based modelling of Dz$\mu$ G depicted in Fig. 2 is further simplified in Fig. 3 with a perspective of equivalent transfer function of DGUs and ESDs. It is categorized into four major categories as system dynamics ($G_{sys}$), energy storage devices ($G_{ESD}$), controllable sources ($G_{con}$), and power variations ($\Delta P_{PV}$, $\Delta P_{W}$). In Fig 3 primary control loop comprises ESDs, while the secondary control loop comprises controllable DGUs. Moreover, SPV and WTG plants with interconnected loads, are considered system-dependent components or disturbances due to power variations. The primary control mechanism involves ESDs for power adjustments, while the secondary control mechanism utilizes controllable DGUs to manage varying load demands^46^. The mathematical formulations of DGUs and ESDs in transfer function form with the nominal value of associated variables with system dynamics are depicted in Table 2.

**Table 2:** Transfer Function of components of Dz$\mu$ with their values^46^.

Distributed Generating Units (DGUs)		
Component	Transfer Function	Parameters
Solar photovoltaic (SPV)^47^	$TF_{PV}(s) = \frac{K_{PV}}{1 + sT_{PV}}$	$K_{PV}=1$, $T_{PV}=1.8$
Wind turbine generator (WTG)^47^	$TF_{WTG}(s) = \frac{K_{WTG}}{1 + sT_{WTG}}$	$K_{WTG}=1$, $T_{WTG}=1.5$
Microturbine generator (MTG)^47^	$TF_{MTG}(s) = \frac{K_{MTG}}{1 + sT_{MTG}}$	$K_{MTG}=1$, $T_{MTG}=1.5$
Biodiesel engine generator (BEG)^23^	$TF_{BEG}(s) \approx \frac{K_{BEG}}{1 + sT_{BEG}}$	$K_{BEG}=1$, $T_{BEG}=0.55$
Biogas turbine generator (BTG)^23^	$TF_{BTG}(s) \approx \frac{K_{BTG}}{1 + sT_{BTG}}$	$K_{BTG}=1$, $T_{BTG}=1.48$
Diesel engine generator (DEG)^47^	$TF_{DEG}(s) = \frac{K_{DEG}}{1 + sT_{DEG}}$	$K_{DEG}=0.003$, $T_{DEG}=2$
Battery energy storage system (BESS)^47^	$TF_{BESS}(s) = \frac{K_{BESS}}{1 + sT_{BESS}}$	$K_{BESS} = -0.003$, $T_{BESS} = 0.1$
Flywheel energy storage system (FESS)^47^	$TF_{FESS}(s) = \frac{K_{FESS}}{1 + sT_{FESS}}$	$K_{FESS} = -0.01$, $T_{FESS} = 0.1$
Ultracapacitor (UC)^48^	$TF_{UC}(s) = \frac{K_{UC}}{1 + sT_{UC}}$	$K_{UC} = -0.7$, $T_{UC} = 0.9$
Electric vehicle (EV)^47^	$TF_{EV}(s) \approx \frac{K_{EV}}{1 + sT_{EV}}$	$K_{EV} = 1$, $T_{EV} = 1$
Inertia and damping^47^	$TF_{Dz\mu G}(s) = \frac{1}{D + sM}$	$D = 0.03$, $M = 0.4$

Figure 3 provides power exchange among various components of the presented model. $\Delta P_{sources}$ is the power produced by controllable sources, $\Delta P_{ESD}$ is the amount of exchangeable power of energy storage devices and $\Delta P_{PV}$, $\Delta P_{W}$ are power produced by PV and WTG units. Frequency deviations ($\Delta f$) occurs due to deviations in $P_{\text {error}}$, which can be expresses as

7 $$\begin{aligned} \Delta f= & \frac{1}{D+Ms}P_{\text {error}} \end{aligned}$$

8 $$\begin{aligned} P_{\text {error}}= & \Delta P_{sources} + \Delta P_{ESD} - P_L \end{aligned}$$

where,

9 $$\begin{aligned} \Delta P_{sources}= & \Delta P_{DEG}+ \Delta P_{MTG}+ \Delta P_{BEG}+\Delta P_{BTG} \end{aligned}$$

10 $$\begin{aligned} P_{ESD}= & \pm \Delta P_{B E S S} \pm \Delta P_{F E S S} \pm \Delta P_{UC} \pm \Delta P_{EV} \end{aligned}$$

$\Delta f$, shown in (7,8) can be minimized by managing $P_{\text {error}}$. Load demand ($P_L$) is supplied with the help of $\Delta P_{sources}$ and $\Delta P_{ESD}$. In load frequency control $\Delta P_{PV}$ and $\Delta P_{W}$ are the power generated by solar and wind power plants, which is utilized in maximum capacity to meet load demand. These powers are stochastic in nature, which initiates a mismatch in power, leading to deviations in frequency. This is the main reason not to include these units in frequency control of the microgrid system.

## Decentralized microgrid approximation model

To maintain frequency stability of $Dz\mu G$ within desired range, it is preferable to obtain an approximated FOPTD model of $Dz\mu G$ system. This is because main model consists of numerous nonlinear and highly complex equations, which leads to inefficient control design. To have an efficient FOPID control design FOPTD model is needed. The mathematical modeling of FOPTD model is depicted in (11).

11 $$\begin{aligned} \phi _{FOPTD}(s)=K\frac{e^{-T_ds}}{1+sT_m} \end{aligned}$$

In (11), $\phi _{FOPTD}(s)$ is the approximated first-order model of $Dz\mu G$, where *K* is steady-state gain, and $T_m$, $T_d$ represents time constant and time delay. Using process reaction curve method^4,46^ method, FOPTD model parameters can be calculated using following equations.

12 $$\begin{aligned} T_d= & 1.3t_1-0.29t_2 \end{aligned}$$

13 $$\begin{aligned} T_m= & 0.67(t_2-t_1) \end{aligned}$$

14 $$\begin{aligned} K= & \frac{\Delta Y_\infty }{U_{step}} \end{aligned}$$

where $\Delta Y_\infty$ is the variation in the output signal when $t=\infty$, and the $U_{step}$ is the amount of variation in input signal. Utilizing process reaction curve and equation (12)- (14), the $Dz\mu G$ is derived to the FOPTD model as shown in (15). Furthermore, identified FOPTD model parameters gain (*K*), time constant ($T_m$), and time delay (*L*) are derived and provided in tabular representation in Table 3.

**Table 3:** Parameters of FOPTD model^46^.

*K*	$T_m$	$T_d$
9.4993	2.2833	0.3951

## Fractional order proportional-integral-derivative controller design

The motivation to use a FOPID controller over a conventional PID lies in its enhanced flexibility and superior control performance. Unlike PID, which uses integer-order integrals and derivatives, FOPID introduces fractional orders ($\lambda$ and $\mu$) that provide additional tuning flexibility, enabling finer control over system dynamics. This extended degree of freedom allows FOPID to better handle complex, nonlinear, or time-delay systems often encountered in microgrids. Further, it offers improved robustness, disturbance rejection, and setpoint tracking across a wider operating range, making it particularly suitable for renewable energy integration and systems with uncertain or varying parameters.

### Design method

**Fig. 4:**
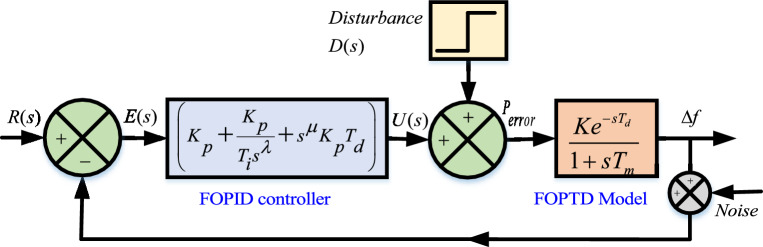
Closed loop system with external load disturbances.

The unity feedback control structure is shown in Fig. 4, which is considered for analyzing the dynamics of FOPTD model depicted in (15) as a plant. Fig. 4, showcased the possible inputs and disturbances which may be occurred in the system at different point.

15 $$\begin{aligned} \begin{aligned} \tilde{\phi _{FOPTD}(s)}=9.4993\frac{e^{-0.3951}}{1+2.2833s} \end{aligned} \end{aligned}$$

**Fig. 5:**
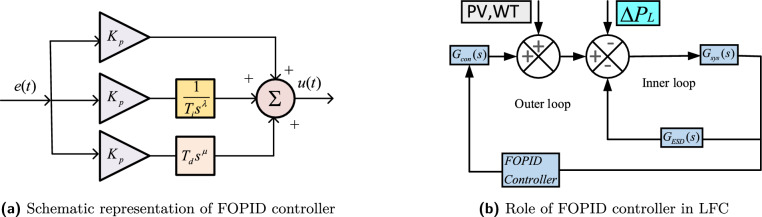
Closed loop based modelling of $Dz\mu G$ components.

FOPID controller is an advanced version of the proportional-integral-derivative (PID) controller. The generalized FOPID architecture is depicted in Fig. 5a. The standard configuration of FOPID contains proportional, derivative, and integral gains, with fractional orders terms of integral ($\lambda$) and derivative ($\mu$). The mathematical formulation of FOPID in the frequency domain is depicted in (16).

16 $$\begin{aligned} U(s)= K_p [1+\frac{1}{T_is^\lambda }+T_ds^\mu ]E(s) \end{aligned}$$

In (16), *U*(*s*) is the FOPID output, $K_p$ is proportional gain, $T_i$ and $T_d$ are integral and derivative variables and *E*(*s*) is error, respectively. The implementation of FOPID controller is to regulate the active powers of controllable source such that load demand can be meet. Schematic diagram of FOPID controller implementation with controllable sources are depicted in Fig. 5b.

The dynamics of the process are described by normalized dead time $\tau$ which is defined as $\tau$ = $\frac{T_d}{T_m}$, where $T_d$ is time delay and $T_m$ is time constant of FOPTD model. The normalized dead time gives a measure of difficulty in controlling the process. In general, the values of the normalized dead time in the range of $0.01 \le \tau \le 4$ is considered^49^. The value of $\tau$ for presented $Dz\mu G$ model is 0.1730. To design efficient controller, fine tuning of FOPID controller is important. To derive suitable gains, the FOPID controller utilizes the ISE and ITSE error indices as performance indices, which are depicted in (17) and (18).

17 $$\begin{aligned} ISE= & \int _{0}^{\infty }|e(t)|^2dt \end{aligned}$$

18 $$\begin{aligned} ITSE= & \int _{0}^{\infty }t|e(t)|^2dt \end{aligned}$$

Minimizing ISE results in reduced overshoot and minimum settling time simultaneously in LDR or SPT control responses. A controller is said to have robustness when it is not sensitive to parameter variations or uncertainties. To improve the robustness of the FOPID controller, the controller has been designed to minimize the ISE and maximize sensitivity as a constraint. The maximum sensitivity ($M_s$) is represented (19) as

19 $$\begin{aligned} M_s=\max _{w\varepsilon [0, \infty ]}\left| \frac{1}{1+G(s)C(s)H(s)} \right| _{s=jw} \end{aligned}$$

Where *G*(*jw*) controller transfer function and *C*(*jw*) plant transfer function. $M_s$ represents the inverse of the shortest distance from the Nyquist curve.

### Tuning of optimal controller

**Fig. 6:**
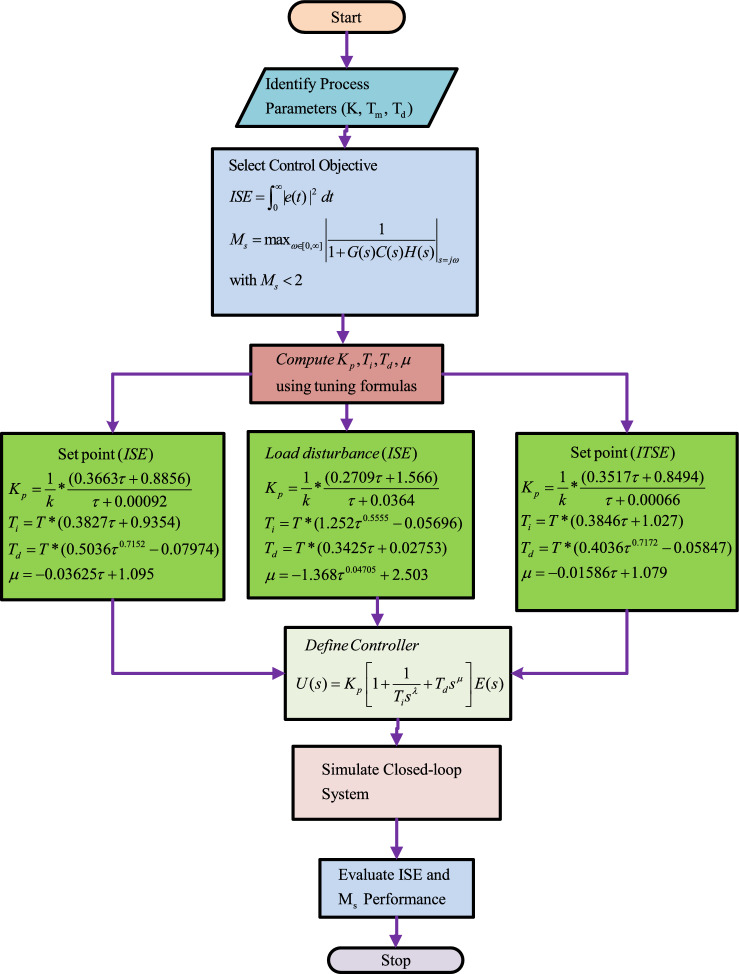
Flowchart of FOPID controller.

To obtain FOPID parameters, the tuning rules of a controller for the control of frequency deviation, Bayat method is preferred and implemented^50^. The set point control and disturbance rejection tasks have been performed separately to minimize the ISE with two values of maximum sensitivity such as $M_s = 1.4$ and $M_s = 2.0$. For $M_s = 1.4$, a system has more robustness, and for $M_s = 2.0$, the aggressiveness is more important. A detailed description of bayat tuning methodology and its implementation in FOPID control design is discussed in Fig. 6.

#### Bayat method: ISE based tuning method

The mathematical formulation of FOPID gains as per Bayat et al^49,51^ are expressed in (20), (21), (22), and (23), respectively.

20 $$\begin{aligned} K_p= & \frac{1}{k}*\frac{(0.3663\tau +0.8856)}{\tau +0.00092} \end{aligned}$$

21 $$\begin{aligned} T_i= & T*(0.3827\tau +0.9354) \end{aligned}$$

22 $$\begin{aligned} T_d= & T*(0.5036\tau ^{0.7152}-0.07974) \end{aligned}$$

23 $$\begin{aligned} \mu= & -0.03625\tau +1.095 \end{aligned}$$

#### Bayat method: ISE-based tuning method for load disturbance

The mathematical formulation of FOPID gains as per Bayat et al^49,51^ are expressed in (24), (25), (26), and (27), respectively.

24 $$\begin{aligned} K_p= & \frac{1}{k}*\frac{(0.2709\tau +1.566)}{\tau +0.0364} \end{aligned}$$

25 $$\begin{aligned} T_i= & T*(1.252\tau ^{0.5555}-0.05696) \end{aligned}$$

26 $$\begin{aligned} T_d= & T*(0.3425\tau +0.02753) \end{aligned}$$

27 $$\begin{aligned} \mu= & -1.368\tau ^{0.04705}+2.503 \end{aligned}$$

#### Bayat method: ITSE-based tuning method

The mathematical formulation of FOPID gains as per Bayat et al^49,51^ are expressed in (28), (29), (30), and (31), respectively.

28 $$\begin{aligned} K_p= & \frac{1}{k}*\frac{(0.3517\tau +0.8494)}{\tau +0.00066} \end{aligned}$$

29 $$\begin{aligned} T_i= & T*(0.3846\tau +1.027) \end{aligned}$$

30 $$\begin{aligned} T_d= & T*(0.4036\tau ^{0.7172}-0.05847) \end{aligned}$$

31 $$\begin{aligned} \mu= & -0.01586\tau +1.079 \end{aligned}$$

Variable $\tau$ can be defined as

32 $$\begin{aligned} \tau =\frac{L}{T} \hspace{2cm} \tau \epsilon [0.1, 4] \end{aligned}$$

### Oustaloup approximation method

To analyze and implement fractional order of integral ($\lambda$) and derivative terms ($\mu$), approximation methods are utilized. These methods approximate fractional-order terms into integer-order equivalents within fixed bandwidth. Numerous approximation methods are available in literature^52^. Oustaloup approximation is a widely accepted method for approximation purposes^53^. The Oustaloup approximation method utilizes the recursive scattering of poles and zeroes to derive equivalent integer order from fractional order. The mathematical representation of fractional term ($s^a$) using the Oustaloup approximation method is given in (33).

33 $$\begin{aligned} s^a \approx K \prod _{m=1}^N \frac{1+\left( s / w_{z, n}\right) }{1+\left( s / w_{p, n}\right) }, a>0 \end{aligned}$$

where, *K* is static gain, and $w_{z,n}$, $w_{p,n}$ denoted frequencies corresponding to zeros and poles, respectively. The Zeros $w_{z,n}$ and poles $w_{p,n}$ are given as in Eqs. (34)

34 $$\begin{aligned} \begin{aligned}&w_{z, n}=w_l\left( \frac{w_h}{w_l}\right) ^{\frac{m+N+(1-r) / 2}{2 n+1}} \\&w_{p, n}=w_l\left( \frac{w_h}{w_l}\right) ^{\frac{m+N+(1-r) / 2}{2N+1}} \end{aligned} \end{aligned}$$

In (34), $w_l$ and $w_h$ represent minimum and maximum limits of frequency of approximation in the range $\omega \epsilon [0.001w_{c}, 1000w_{c}]$ such that $w_lw_h = 1$. In simulation, the designed FOPID controller is approximated using Oustaloup method. The frequency range considered for approximation is 0.001–1000 rad/sec with approximation order of 5.

## Results and discussion

### Frequency response without controller

**Fig. 7:**
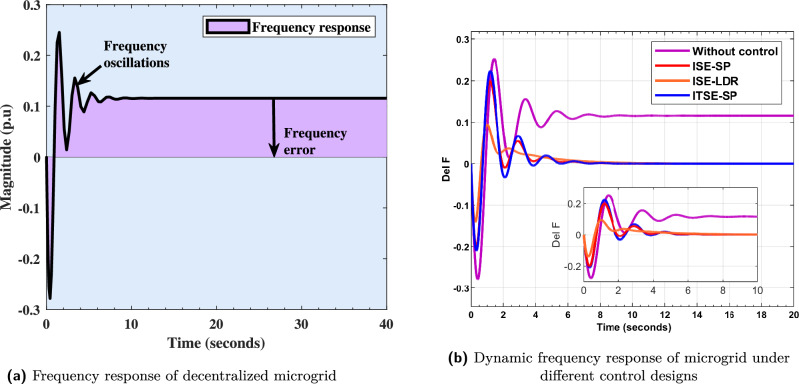
Frequency response of decentralized microgrid with and without FOPID controller.

Before implementing a controller, it is essential to analyze the frequency response of microgrid model. The frequency response of $\hat{G_{Dz\mu G}(s)}$ model is illustrated in Fig. 7a. This response can be evaluated using standard transient performance indicators such as rise time (0.1698 s), settling time (8.0089 s), peak time (0.6222 s), peak value (0.4673 pu), undershoot (258.5687), and overshoot (80.9992). The system exhibits significant overshoot and undershoot, along with underdamped oscillations, with offset error of 0.1803 pu. Such deviations are undesirable, as the desired frequency deviation is zero. In this contribution, $\Delta f$ is the control parameter which is to be minimized through FOPID controller.

To further assess the system performance, various error indices have been computed. The integral absolute error (IAE) is 9.182, the integral time absolute error (ITAE) is 225.9, the integral squared error (ISE) is 1.756, and the integral time squared error (ITSE) is 40.9. These values highlight the poor dynamic performance of the system . However, these deficiencies evident in both transient response and error indices can be effectively mitigated by integrating a FOPID controller into the $G_{Dz\mu G}$ model.

### Frequency response with FOPID controller

To suppress the deviations and eliminate the offset error of the frequency response, various variants of the FOPID controller are designed. These controllers are identified as FOPID-$Bayat_{SP1.4}$, FOPID-$Bayat_{SP2.0}$, and FOPID-$Bayat_{LD1.4}$. These controller designs are formulated using the tuning methodology i.e. Bayat method for SPT and LDR scanerios with maximum sensitivity ($M_s=1.4$ and $M_s=2.0$). The gains of designed FOPID controllers are obtained using Bayat method for both modes, which are given in Table 4. The mathematical formulation of all designed FOPID controllers are presented in (35)-(37).

**Table 4:** FOPID controller gains for frequency regulation of $\hat{G_{Dz\mu G}(s)}$ Model.

Gains	FOPID-$Bayat_{SP1.4}$	FOPID-$Bayat_{SP2.0}$	FOPID-$Bayat_{LD1.4}$
$K_p$	1.5596	2.9959	1.4967
$T_i$	0.8717	0.4651	0.9488
$T_d$	0.1012	0.1022	0.0853
$\lambda$	1	1	1
$\mu$	1.0850	1.2154	1.0767

$$\begin{aligned} C_{\text {FOPID-Bayat}_{\text {SP1.4}}}(s)= & \frac{1.35950332\,s + 1.5596 + 0.137581735984\,s^{2.0850}}{0.8717\,s}\\ C_{\text {FOPID-Bayat}_{\text {SP2.0}}}(s)= & \frac{1.39339309\,s \;+\; 2.9959 \;+\; 0.142404773798\,s^{2.2154}}{0.4651\,s}\\ C_{\text {FOPID-Bayat}_{\text {LD1.4}}}(s)= & \frac{1.41058096\,s \;+\; 1.4867 \;+\; 0.120322555888\,s^{2.076}}{0.9488\,s} \end{aligned}$$

35 $$\begin{aligned} C_{FOPID-Bayat_{SP1.4}}= & 1.5596\left[ 1+\frac{1}{0.8717s^1}+0.1012s^{1.0850}\right] \end{aligned}$$

36 $$\begin{aligned} C_{FOPID-Bayat_{SP2.0}}= & 2.9959 \left[ 1+\frac{1}{0.4651s^1}+0.1022s^{1.2154}\right] \end{aligned}$$

37 $$\begin{aligned} C_{\text {FOPID-Bayat}_{\text {LD1.4}}}= & 1.4867 \left[ 1 + \frac{1}{0.9488\, s^{1}} + 0.0853\, s^{1.076} \right] \end{aligned}$$

In (35)-(37), $C_{FOPID-Bayat_{SP1.4}}$, $C_{FOPID-Bayat_{S2.0}}$, $C_{FOPID-Bayat_{LD1.4}}$, are FOPID controller transfer functions for SPT and LDR modes, respectively. All variants of Bayat-tuned FOPID controllers ($C_{FOPID-Bayat_{SP1.4}}$, $C_{FOPID-Bayat_{SP2.0}}$, and $C_{FOPID-Bayat_{LD1.4}}$), are implemented to eliminate deviations occurring in the frequency response of the model. The dynamic frequency response of the microgrid with the controllers discussed is discussed in Fig. 7b. From figure it is clear that, all discussed controllers mitigate the offset error and maintain a frequency stability with improved transient response. 8.

### Stability analysis

**Fig. 8:**
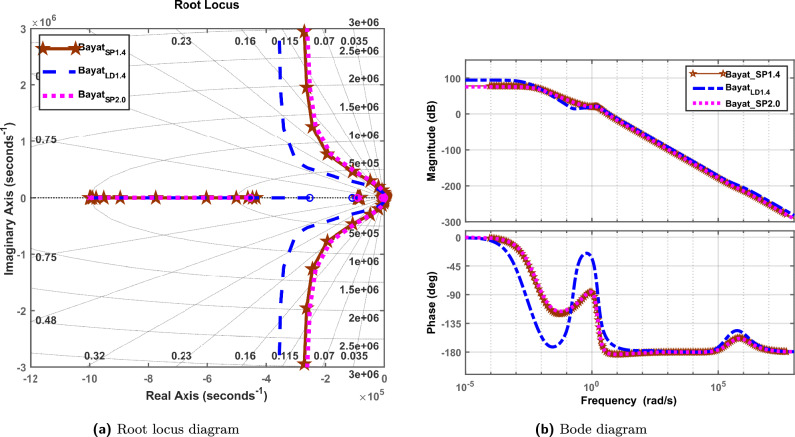
Root locus and bode diagram.

To ensure stability of the microgrid and its control designs, root locus plot and bode plots are provided in Fig. 8a and b. For this study, the frequency domain stability analysis is carried out through bode plots to validate the stability performance of the proposed control scheme. Figure 8 provides a comparative analysis of the closed-loop dynamics for three Bayat-tuned FOPID controllers—Bayat*SP*1.4, Bayat*LD*1.4, and Bayat$_{SP2.0}$—through root locus and Bode plots. Fig. 8a presents the root locus diagram, illustrating the pole migration paths as system gain varies. It is evident that all three controllers shift the dominant poles toward the left half of the s-plane, indicating enhanced system stability. Among them, Bayat*SP*1.4 and Bayat*LD*1.4 exhibit superior damping characteristics with poles closer to the real axis, resulting in a faster and more stable response. Bayat$_{SP2.0}$ shows comparatively less damping, with some poles near the imaginary axis, suggesting higher oscillatory behavior under dynamic conditions. Fig. 8b shows the Bode diagram, providing insight into the frequency response characteristics of the controllers. In the magnitude plot, all designs maintain adequate gain margins across the frequency range, ensuring robust performance. The phase response reveals that Bayat*SP*1.4 and Bayat*LD*1.4 maintain better phase stability over the mid-frequency band compared to Bayat*SP*2.0, which exhibits a pronounced phase dip. These observations collectively affirm that Bayat*SP*1.4 and Bayat$_{LD1.4}$ offer better control authority, phase margin, and robustness to dynamic variations in the microgrid system. Eigenvalues of FOPID-$Bayat_{SP1.4}$,FOPID-$Bayat_{SP2.0}$, and FOPID-$Bayat_{LD1.4}$ controllers are −1.0000 + 0.0000i, −0.0811 + 0.0000i, −0.0066 + 0.0000i, −0.0005 + 0.0000i, −0.0001 + 0.0000i, −0.0000 + 0.0000i.

In this contribution, three versions of FOPID controller, i.e., $C_{FOPID-Bayat_{SP1.4}}$, $C_{FOPID-Bayat_{SP2.0}}$, and $C_{FOPID-Bayat_{LD1.4}}$ are discussed for frequency control of microgrid. A comparative analysis is performed among the discussed FOPID controller, and their performances are analyzed in terms of convergence speed, frequency stability, error indices, and control effort for cases which are mention here as

- Case 1. Random load disturbances.
- Case 2. Solar power variations.
- Case 3. Wind power variations.
- Case 4. Parameter variation.
- Case 5. Multiple disturbances test case.
- Case 6. Real- time validation.

### FOPID controller design and validation for random load disturbance

**Fig. 9:**
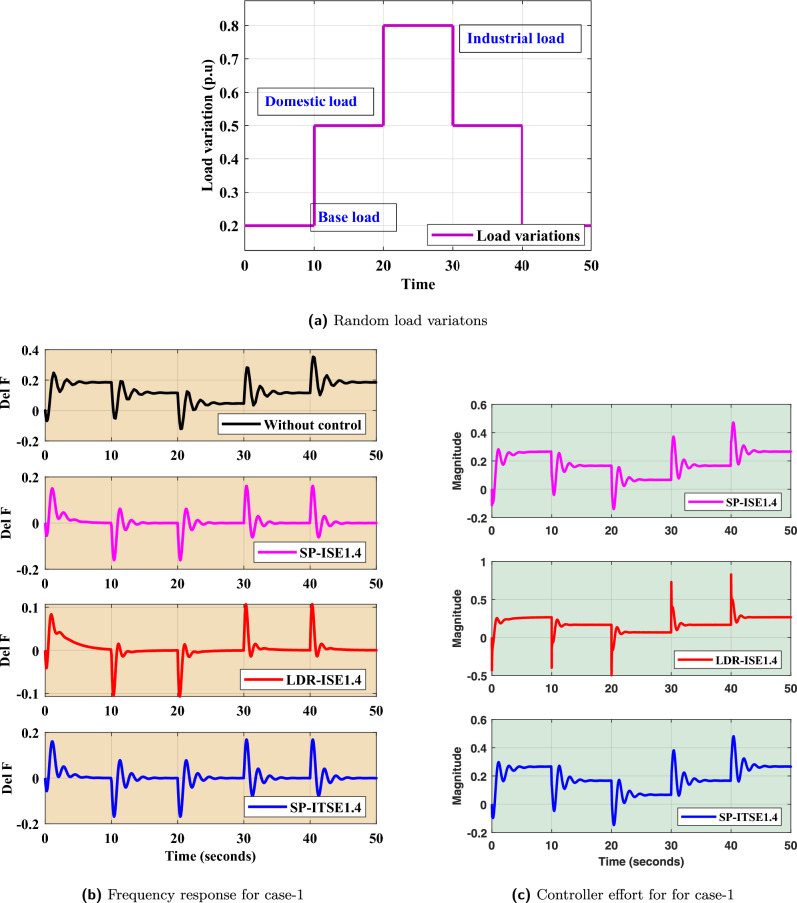
Case 1: Random load disturbances.

In this study, a scenario involving random load variations is analyzed to evaluate the performance of the proposed control strategy. Specifically, the system is subjected to a composite loading profile that includes a base load of 0.2 per unit (p.u), a domestic load component of 0.6 p.u, and an industrial load of 0.8 p.u. These variations aim to simulate realistic demand conditions that a $Dz\mu G$ model may encounter during its operation. In this contribution, considered penetration levels of wind, solar, Biogas, and Biodiesel are 50%, 50%, 30%, and 25%, respectively. The composite profile of these load changes is illustrated in Fig. 9a, which shows the sequence and magnitude of the applied load steps over time.

The frequency response due to FOPID-$Bayat_{SP1.4}$, FOPID-$Bayat_{SP2.0}$, and FOPID-$Bayat_{LD1.4}$ for multiple load variations is presented in Fig. 9b. As observed from the figure, the proposed FOPID controller eliminated offset errors with improved transient performance. Transient response indicators of frequency response corresponding to this case are mentioned in Table 5. This includes reduced overshoot, faster settling time, and better frequency regulation during abrupt load transitions.

Furthermore, the controller effort, which reflects the signal required to maintain system stability and performance for FOPID-$Bayat_{SP1.4}$, FOPID-$Bayat_{SP2.0}$, and FOPID-$Bayat_{LD1.4}$ for multiple load variations, is shown in Fig. 9c. It is evident from this figure that the Bayat-tuned FOPID controller not only enhances the dynamic response but also operates more efficiently by minimizing excessive control actions. This balance between performance and control effort underscores the effectiveness of the proposed controller.

### FOPID controller design and validation for solar power variations

**Fig. 10:**
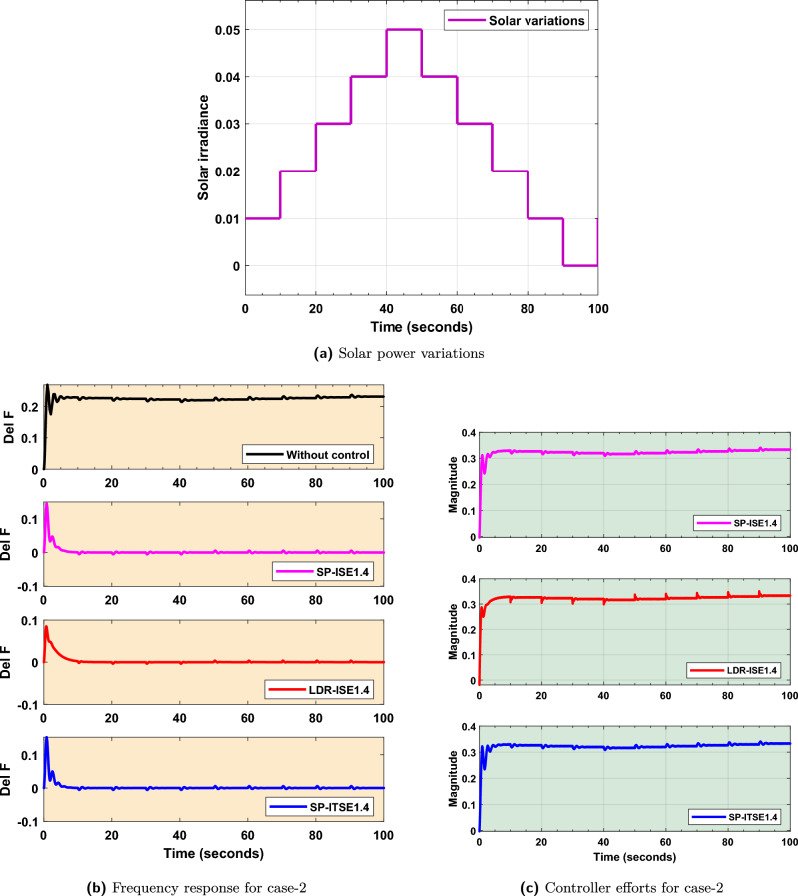
Case 2. Solar power variations.

The variation in solar power generation over the course of a typical day is illustrated in Fig. 10a. In this contribution, the penetration levels of solar are considered to be 50%. This profile represents the natural fluctuation in solar irradiance due to factors such as the sun’s position and environmental conditions. These variations introduce disturbances to the microgrid. To assess the FOPID-$Bayat_{SP1.4}$, FOPID-$Bayat_{SP2.0}$, and FOPID-$Bayat_{LD1.4}$ controller’s capability to maintain frequency stability under solar variations, frequency response is presented in Fig. 10b. The results indicate that the Bayat-tuned FOPID controllers effectively mitigates the adverse effects of solar intermittency. A quantitative assessment of the frequency response for solar power variations is summarized in Table 5. This table concludes that the FOPID-$Bayat_{SP1.4}$, FOPID-$Bayat_{SP2.0}$, and FOPID-$Bayat_{LD1.4}$ control designs provide better oscillation damping, faster stabilization, and reduced frequency deviations.

Additionally, the control efforts required by the various controllers to sustain frequency stability during the solar power variations are shown in Fig. 10c. From this figure, it is clear that the proposed FOPID controller not only achieves enhanced dynamic performance but require relatively lower control effort, highlighting its efficiency and robustness.

**Fig. 11:**
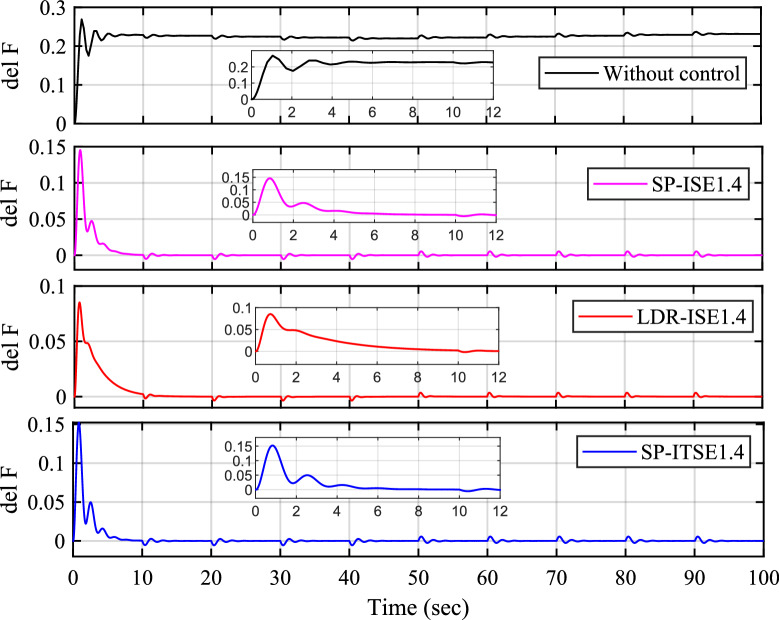
Frequency response for case-2.

### FOPID controller design and validation for wind power variations

**Fig. 12:**
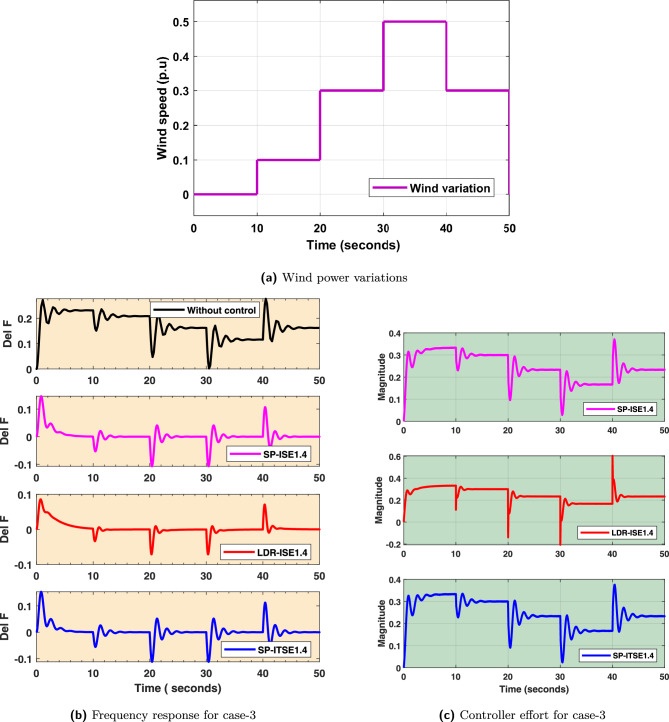
Case 3. Wind power variations.

The variation in wind power generation, which inherently results from the stochastic and intermittent nature of wind speed, is illustrated in Fig. 12a. In this contribution, considered penetration levels of wind are 50%. These fluctuations are a common challenge in microgrid operation, as they introduce significant disturbances to system frequency and demand effective control mechanisms to ensure stable operation. The frequency response of $Dz \mu G$ model under the influence of multiple load variations is shown in Fig. 12b. FOPID-$Bayat_{SP1.4}$, FOPID-$Bayat_{SP2.0}$, and FOPID-$Bayat_{LD1.4}$ controllers provide improved frequency regulation, reduced overshoot, and faster settling during this case. -The control efforts of FOPID-$Bayat_{SP1.4}$, FOPID-$Bayat_{SP2.0}$, and FOPID-$Bayat_{LD1.4}$ controllers to maintain frequency stability during wind variations are presented in Fig. 12c. As seen from figure, the FOPID-$Bayat_{SP1.4}$, FOPID-$Bayat_{SP2.0}$, and FOPID-$Bayat_{LD1.4}$ controllers maintains system stability with comparatively lower control magnitude, emphasizing its efficiency and robustness in the presence of high variability. Further, efficacy of FOPID-$Bayat_{SP1.4}$, FOPID-$Bayat_{SP2.0}$, and FOPID-$Bayat_{LD1.4}$ controllers are analysed through error indices, which are mentioned in Table 6. Additionally, a statistical analysis of the frequency response during the wind power variation scenario is included in Table 5 to prove the efficacy of control designs.

###  Multiple disturbances test case

**Fig. 13:**
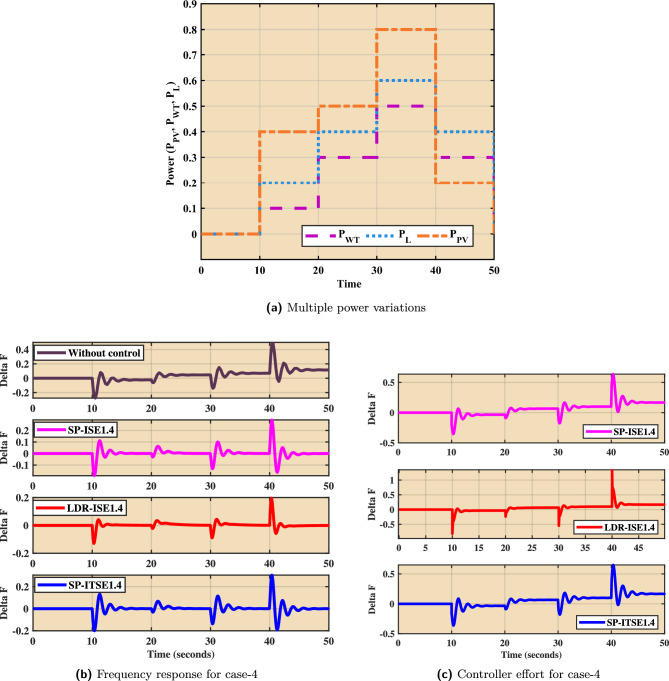
Case 4. Multi-disturbance test case.

The impact of multiple power variations from wind turbine (WT), photovoltaic (PV), and load ($P_L$) sources—is illustrated in Fig. 13. Figure 13a displays the normalized power variation profiles of $P_{WT}$, $P_{PV}$, and $P_{L}$ over time, reflecting a typical scenario with high variability and intermittent renewable inputs. This dynamic environment poses a significant challenge to microgrid frequency stability and requires robust control strategies to maintain operational resilience. Figure 13b presents the frequency response of the $Dz\mu G$ system for Case-4 under the influence of these variations. In the absence of control, the system exhibits pronounced frequency deviations, confirming the destabilizing effect of renewable intermittency and load fluctuations. In contrast, the implementation of fractional-order PID (FOPID) controllers—namely SP-ISE1.4, LDR-ISE1.4, and SP-ITSE1.4—significantly improves frequency regulation. These controllers effectively reduce frequency deviation, enhance damping, and achieve quicker settling times. The associated control efforts are shown in Fig. 13c. All three controllers demonstrate adequate actuation with reasonable control signal magnitudes, ensuring frequency stabilization without introducing excessive control action. Among them, SP-ISE1.4 and SP-ITSE1.4 exhibit smoother transitions, suggesting better performance under the fluctuating power conditions of Case-4. This validates the proposed FOPID control designs as robust and efficient solutions for maintaining microgrid frequency stability in the presence of diverse and time-varying disturbances.

### Frequency analysis under stochastic load

**Fig. 14:**
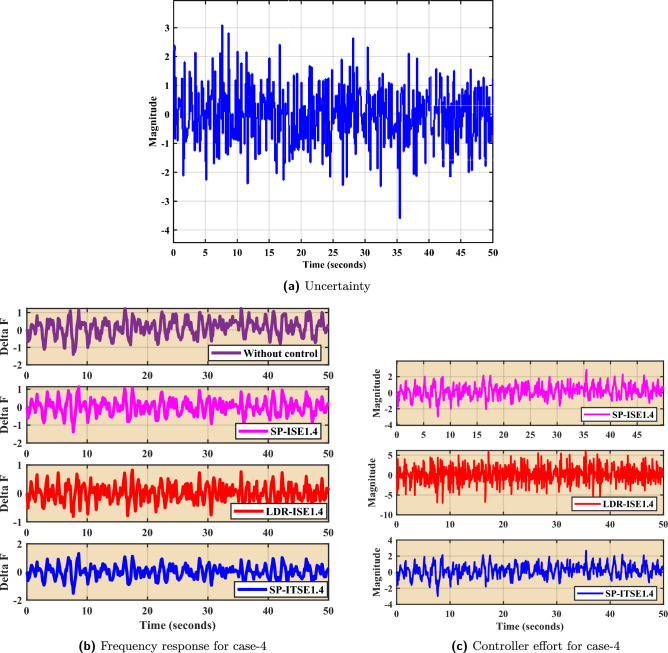
Frequency analysis under stochastic load.

Figure 14 illustrates the performance of the proposed control strategies under a stochastic load disturbance scenario. Figure 14a depicts the randomly varying power input profile used to emulate real-world uncertainties and rapid fluctuations in load demand. Such stochastic variations pose significant challenges to microgrid frequency stability and demand resilient control designs. The corresponding frequency responses for Case-4 are shown in Fig. 14b. In the absence of control, the system experiences large and sustained frequency deviations, reflecting poor disturbance rejection capability. In contrast, the application of FOPID controllers—SP-ISE1.4, LDR-ISE1.4, and SP-ITSE1.4—leads to substantial improvements in frequency regulation. These controllers successfully mitigate the impact of stochastic disturbances, as seen by reduced oscillation magnitudes and enhanced damping behavior. Figure 14c presents the associated control efforts for each controller. Despite the erratic nature of the load input, all three controllers maintain stable and bounded control signals. Notably, SP-ITSE1.4 demonstrates the most efficient control effort with reduced actuation magnitudes, indicating better control economy and robustness. The overall performance confirms the effectiveness of the proposed Bayat-tuned FOPID controllers in preserving microgrid frequency stability under highly uncertain and non-deterministic load conditions.

**Table 5:** Statistical analysis of the proposed controllers.

Controller’s		FOPID-$Bayat_{SP1.4}$	FOPID-$Bayat_{SP2.0}$	FOPID-$Bayat_{LD1.4}$
Scenario’s	Specifications			
Case 1	$T_r$	$4.99e-5$	$6.31e-4$	$1.79e-4$
	$T_p$	30.3985	40.3116	30.4056
	$\%O_s$	5.136e5	4.9531e4	1.5077e5
	$T_s$	49.8586	49.9534	49.9330
	Controllability	Yes	Yes	Yes
	control effort	0.285	0.25	0.295
Case 2	$T_r$	4.0219e-5	2.2123e-4	1.2179e-4
	$T_p$	0.8353	.7316	0.8327
	$\%O_s$	1.14e7	5.2163e4	3.899e6
	$T_s$	49.8498	49.9552	49.9315
	Controllability	Yes	Yes	Yes
	control effort	0.32	0.33	0.353
Case 3	$T_r$	6.4248e-5	0.0087	2.3681e-4
	$T_p$	0.8186	0.7161	0.8153
	$\%O_s$	5.3477e5	6.1249e4	1.5181e5
	$T_s$	49.8615	49.9535	49.9331
	Controllability	Yes	Yes	Yes
	control effort	0.33	0.32	0.34
Case 4	$T_r$	6.4248e-5	0.0308	3.3681e-4
	$T_p$	0.7186	0.6161	0.5153
	$\%O_s$	5.4547e5	6.179e4	1.581e5
	$T_s$	49.615	49.535	49.331
	Controllability	Yes	Yes	Yes
	control effort	0.17	0.12	0.15

**Table 6:** Comparison of different error indices for various FOPID tuning rules.

**Error Index**	**FOPID-**$Bayat_{SP1.4}$	**FOPID-**$Bayat_{SP2.0}$	**FOPID-**$Bayat_{LD1.4}$
IAE	9.336	**8.737**	15.180
ITAE	223.5	**223.0**	280.0
ITSE	41.86	**40.39**	98.20
ISE	2.318	**1.706**	8.029

### Sensitivity analysis

In this study, sensitivity analysis is performed considering parametric variations in time constant of $T_{bdeg}$, $T_{bgtg}$, as these are the more sensitive component of the presented model. A variation of $\pm 50$ in the range of $25\%$ i.e., (−50,−25,+25,+50) is considered for the analysts purpose. Moreover, same variation is applied to damping (*D*) and inertia constant (*M*) of model to study behavioral changes of $Dz \mu G$ model due the these variations. Statistical analysis based on the parametric variation is provided in Table 7. Moreover, impact of these parameters on $\Delta f$ is also depicted in Table 7. Additionally, the impact of parametric analysis are understood by transient response indicators i.e., $T_s$, $T_p$, $T_r$, and various error indices such as IAE, ITAE, ISE, ITSE.

**Table 7:** Parametric variations.

	(%)	*Ts*(*s*)	*Tp*(*s*)	*Tr*(*s*)	IAE	ITAE	ISE	ITSE	$\Delta f$
$T_{bdeg}$	−50	9.9687	.8272	6.9789	0.02106	0.2419	0.01597	.4898	.00042
	−25	9.9687	0.8213	0.0099	0.02091	0.2419	0.1698	0.4787	.0004203
	+25	9.9674	.8503	0.0110	0.02285	.2419	0.02016	.4566	.0004848
	+50	9.9758	.8663	.0115	.02418	.2418	.02209	.445	.0005121
$T_{bgtg}$	−50	9.9665	.7850	.0137	.01994	.2415	.01508	.5228	.0006433
	−25	9.9681	.8140	.0122	.0206	.2416	.01684	.4946	.0005908
	+25	9.9786	.8505	.0060	.02253	.2423	.01978	.4429	.0002269
	+50	9.9930	.8630	9.7485e4	.02357	.2429	.02097	.4208	−2.928e-5
*D*	−50	9.9739	0.8353	0.0099	.02159	.2419	.01853	.4669	.0004178
	−25	9.9734	0.8349	0.0099	.02155	.2419	.01847	.4673	.0004182
	+25	9.9726	0.8354	0.0099	.02148	.2419	.01835	.4681	.0004191
	+50	9.9723	0.8353	0.0090	.02145	.2419	.01832	.4686	.0004195
*M*	−50	9.9686	0.6133	0.0058	.01907	.2419	.01677	.4675	.0004262
	−25	9.9693	0.7331	0.0080	.02021	.2419	.01762	.4676	.0004221
	+25	9.9591	0.9237	0.0106	.02299	.2419	.01916	.4678	.0003694
	+50	9.9793	1.0043	0.0154	.02461	.2419	.0189	.4680	.0005122

### Real-time validation

**Fig. 15:**
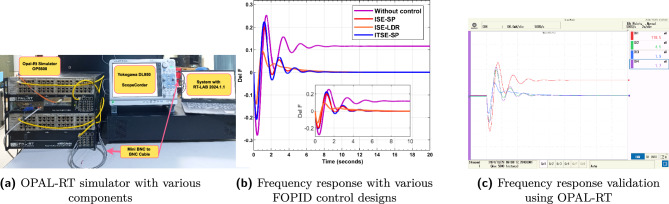
Real time validation.

To validate the real-time performance of the discussed version of FOPID controller, OP5600 Opal-RT real-time simulator is used, which is depicted in Fig. 15a. It is a high-performance platform equipped with a 12-core processor, ideal for complex real-time simulations. The system includes OP5142 analog output cards, which ensure accurate signal transfer to external devices. For output visualization, the simulator is connected to a Yokogawa DL950 oscilloscope via Mini-BNC to BNC cables, enabling direct monitoring and capture of signals from the simulator’s output port. Further, a real-time validation procedure using OPAL-RT is provided in algorithm 1. Furthermore, the experimental setup details are as follows,

- Sampling frequency: 20 kHz
- Signal scaling for analog/digital interfaces
- Use of RT-LAB’s scaling blocks and anti-aliasing filters
- Latency compensation: No explicit compensation used due to low-latency (<1 ms) setup; this is now noted and justified

Various version of FOPID controllers are tested on OPAL-RT simulator to test the feasibility of control design and their effectiveness in maintaining frequency responses of $Dz \mu G$ model. The frequency response of $Dz \mu G$ model using various FOPID controllers via MATLAB without considering delay is provided in Fig. 15b. The OPAL-RT based frequency response of various FOPID controllers is depicted in Fig. 15c. Based on Fig. 15b and c, it is observed that frequency responses are equivalent, which justify the FOPID control designs.

**Algorithm 1:**
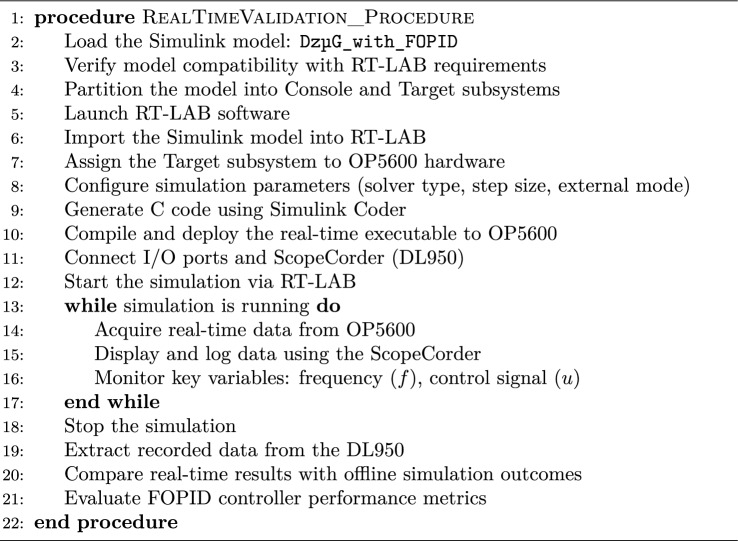
Real-Time Validation Procedure Using OPAL-RT

## Conclusion

This article investigates the Bayat method for frequency stability of a decentralized microgrid model by designing FOPID controllers. To implement FOPID controller, a linear transfer function of $Dz \mu G$ model is obtained. To approximate the transfer function of $Dz \mu G$ model, it is linearized and approximated as FOPTD model. FOPID control design is derived for set point tracking and load disturbance rejection modes. A comparative assessment of variants of FOPID controllers is performed to ascertain the suitable control design to maintain frequency stability of decentralized microgrids. Among the utilized tuning methods such as FOPID-$Bayat_{SP1.4}$, FOPID-$Bayat_{SP2.0}$, and FOPID-$Bayat_{LD1.4}$. FOPID-$Bayat_{SP2.0}$ emerges as a suitable and efficient control design for frequency error mitigation of the $Dz \mu G$ model. The results showcased in this study highlight the efficacy of the FOPID-$Bayat_{SP2.0}$ method concerning transient response, control signal magnitude, and frequency deviation plots. The statistical and error index analyses validate the superiority of the proposed controllers across all scenarios. All *FOPID–Bayat* configurations maintained controllability, with rise times as low as $4.02 \times 10^{-5}\,\text {s}$ and settling times remaining stable around $49.8\,\text {s}$. The control effort was minimized to 0.12, indicating improved efficiency. In addition, *FOPID–Bayat*$_{SP2.0}$ demonstrated the best error performance, with minimum IAE (8.737), ITAE (223.0), ITSE (40.39), and ISE (1.706). These findings establish *FOPID–Bayat*$_{SP2.0}$ as the most effective configuration for achieving robust frequency stability and reduced dynamic errors. Additionally, tabulated data of tuning parameters and time domain specifications of model validate the FOPID control designs for frequency stability of $Dz \mu G$ model.

The proposed Bayat-driven FOPID controller design has several limitations. Its tuning is system-specific and may not generalize well to other microgrid configurations without re-tuning. The reliance on reduced-order models introduces performance sensitivity to modeling inaccuracies. While OPAL-RT real-time validation adds value, it does not fully capture physical nonlinearities or communication issues encountered in practical deployment. Moreover, the study does not explore the controller’s robustness under extreme disturbances or compare it extensively with other optimization techniques. Finally, economic and cybersecurity aspects, crucial for real-world microgrid applications, remain unaddressed.

Looking ahead, future endeavors could integrate advanced control techniques to tune FOPID controllers using learning-based optimization approaches. Moreover, the cascaded based control design could be explored as an extension of this work to maintain frequency stability of the $Dz \mu G$ model. Another promising avenue for extending this work involves addressing the cybersecurity issues associated with various control strategies and enhancing the resilience of microgrids. Further, the efficacy of the FOPID controller is tested for probabilistic profiles of solar and wind power in the future.

## Data Availability

All data generated or analysed during this study are included in this published article.

## Abbreviations

Dz$\mu$G: Decentralized microgrid
BEG: Biodiesel engine generator
BTG: Biogas turbine generator
BESS: Battery energy storage system
DGUs: Distributed generation units
DEG: Diesel engine generator
ESDs: Energy storage devices
EVs: Electric vehicles
FESS: Flywheel energy storage system
FOPTD: First-order plus time delay
FOPID: Fractional order proportional-integral-derivative
LDR: Load disturbance rejection
LFC: Load frequency control
MTG: Microturbine generator
RESs: Renewable energy sources
SPT: Set-point tracking
SPV: Solar photovoltaic
UC: Ultracapacitor
WTG: Wind-turbine generator

## List of symbols

*M*: Inertia constant
*D*: Damping constant
FOPID-Bayat$_{SP1.4}$: FOPID for 1.4 SPT
FOPID-Bayat$_{SP2.0}$: FOPID for 2.0 SPT
FOPID-Bayat$_{LD1.4}$: FOPID for 1.4 LDR
$G_{\text {sys}}$: Transfer function of dynamics
$G_{\text {con}}$: Transfer function of controllable sources
$G_{\text {ESD}}$: Transfer function of ESD
$G_{\text {inner}}$: Transfer function of inner loop
$C_{\text {FOPID-Bayat}_{SP1.4}}$: Controller for SP1.4
$C_{\text {FOPID-Bayat}_{SP2.0}}$: Controller for SP2.0
$C_{\text {FOPID-Bayat}_{LD1.4}}$: Controller for LD1.4
$G_{\text {Dz}\mu \text {G}}$: Transfer function of decentralized microgrid
$P_{\text {error}}$: Net power error
$$P_L$$: Load power
$$\Delta f$$: Frequency deviation
$P_{\text {PV}}, P_{\text {WT}}$: Powers of PV and WT
*K*, $$T_d$$, $$T_m$$: FOPTD parameters
$P_{\text {BESS}}, P_{\text {FESS}}, P_{\text {UC}}, P_{\text {EV}}$: Power of BESS, FESS, UC, EV
$P_{\text {DG}}, P_{\text {MT}}, P_{\text {BEG}}, P_{\text {BTG}}$: Powers of DG, MT, BEG, BTG
